# Fungal Endophthalmitis in a Case of Rhino-Orbito-Cerebral Mucormycosis: Successfully Treated With Amphotericin B Colloidal Dispersion

**DOI:** 10.3389/fmicb.2022.910419

**Published:** 2022-06-15

**Authors:** Yinlong Zhao, Wenbin Tian, Jiankai Yang, Xueqing Li, Huaihai Lu, Ning Yu, Pei Zhang, Chao Liu, Pengfei Chen, Guang Lei, Ya Liu

**Affiliations:** ^1^Department of Anesthesiology and Intensive Care, The Second Hospital of Hebei Medical University, Shijiazhuang, China; ^2^Department of Neurosurgery, The Second Hospital of Hebei Medical University, Shijiazhuang, China; ^3^Department of Ophthalmology, The Second Hospital of Hebei Medical University, Shijiazhuang, China; ^4^Department of Otolaryngology, The Second Hospital of Hebei Medical University, Shijiazhuang, China

**Keywords:** mucormycosis, fungal infections, rhino-orbito-cerebral, amphotericin B colloidal dispersion, topical treatment

## Abstract

**Background:**

Rhino-orbito-cerebral mucormycosis (ROCM) is an acute, fulminant, opportunistic fungal infection that usually occurs in diabetes or immunocompromised patients. Amphotericin B combined with surgical debridement remains the standard treatment, although it is controversial due to its lager nephrotoxicity. Thus far, no studies have reported the treatment for ROCM-associated fungal endophthalmitis because the exact pathogenesis and transmission routes in ROCM remain unclear. Here, we reported a case of ROCM complicated with fungal endophthalmitis treated favorably with amphotericin B colloidal dispersion (ABCD) in combination with other antifungals and surgical debridement.

**Case Presentation:**

A 34-year-old woman with diabetes was admitted to our hospital owing to right-sided headache for 8 days, blindness with swelling in the right eye for 5 days, and blindness in the left eye for 1 day. MRI showed that the patient had sphenoid sinus, sinuses, frontal lobe lesions, and proptosis of the right eye. Metagenomic sequencing revealed that the patient had *Rhizopus oryzae* infection. During hospitalization, the patient received intravenous ABCD, oral posaconazole, and topical amphotericin B and underwent surgical debridement. After 67 days of treatment, the patient’s condition was significantly improved, and limb muscle strength showed grade V. *Rhizopus oryzae* showed negative results, and conjunctival swelling decreased. Additionally, no nephrotoxicity occurred during treatment. After discharge, the patient’s treatment was transitioned to oral posaconazole and she was free of complaints during the 30-day follow-up without any additional treatment for ROCM.

**Conclusion:**

Treatment with ABCD combined with other antifungal drugs and surgical debridement for ROCM complicated with fungal endophthalmitis showed remarkable efficacy and good safety. Hence, this regimen is a promising treatment strategy for this fatal disease.

## Introduction

Mucormycosis is a rapidly progressive and fatal disease caused by fungal infection, with an annual incidence of 1.2/1,000,000 people (Cornely et al., 2014). Pathogenic microorganisms are from the *Mucoraceae* family, which typically involve *Mucor*, *Rhizopus*, and *Absidia* species (Baldin and Ibrahim, 2017). Immunocompromised patients, especially those with poorly controlled diabetes, reportedly have the highest risk of developing mucormycosis (Kashyap et al., 2019). Such infections are usually manifested as rhino-orbito-cerebral (ROC), pulmonary, cutaneous, gastrointestinal, disseminated, and uncommon presentation in form, of which ROC is the most common form (Petrikkos et al., 2012). The exact pathogenesis and transmission routes of ROC mucormycosis (ROCM) are unclear. It is generally believed that the fungus initially infects the nasal mucosa and then spreads to the paranasal sinuses, orbits, and finally to the intracranial fossa (Spellberg et al., 2005).

The main approaches to mucormycosis treatment include reversal of the patient’s immunocompromised state, aggressive treatment with systemic antifungals, and surgical debridement (Bellazreg et al., 2015). A previous study reported that amphotericin B can combine with ergosterol in the fungal cell membrane and, consequently, alter the permeability of the fungal cell membrane, resulting in fungal death; however, its usage is limited, as it generally causes nephrotoxicity (Guimarães and Moura, 2022). Therefore, alternative treatments and novel drugs should be considered.

At present, there are three amphotericin B dosage forms in China: traditional amphotericin B, amphotericin B colloidal dispersion (ABCD), and liposomal amphotericin B. Domestic liposomal amphotericin B is different from liposomal amphotericin B abroad and shows no significant improvement in nephrotoxicity compared with amphotericin B. Therefore, in this sense, ABCD is currently the only amphotericin B lipid formulation in China whose nephrotoxicity is significantly lower than that of traditional amphotericin B. ABCD is a colloidal dispersion of disc-shaped nanoparticles formed by amphotericin B and cholesteryl sulfate in a molecular molar ratio of 1:1. Cholesteryl sulfate can combine with amphotericin B and reduce the binding of amphotericin B to cholesterol in human cell membranes, thereby significantly reducing the nephrotoxicity (Herbrecht et al., 2001). Moreover, after being absorbed by the organs with reticuloendothelial system, such as liver, spleen, and lung, it will form a temporary reservoir, and then be dissociated in phagocytes of reticuloendothelial system to release free amphotericin B, so avoiding the higher serum concentrations associated with amphotericin B and lead to lower nephrotoxicity (Oppenheim et al., 1995). For example, in infected brain tissue, the mean concentration of ABCD was significantly higher than that of conventional amphotericin B (Groll et al., 2000). Foreign study has also shown that ABCD and liposomal amphotericin B have the same curative effect (Clemons and Stevens, 1998). Besides, the efficacy and safety of ABCD therapy has also been demonstrated in patients with pre-existing renal insufficiency, development of nephrotoxicity during amphotericin B therapy, or mucormycosis ineffective against amphotericin B (Herbrecht et al., 2001). However, to the best of our knowledge, there has been no report regarding the treatment for ROCM-associated fungal endophthalmitis.

In this report, we describe a case of refractory ROCM complicated with fungal endophthalmitis that was successfully treated with ABCD in combination with other antifungals and surgical debridement. This study was approved by ethics committee of The Second Hospital of Hebei Medical University. Written informed consent was obtained from the individual for the publication of any potentially identifiable images or data included in this article.

### Case Presentation

**Figure 1:**
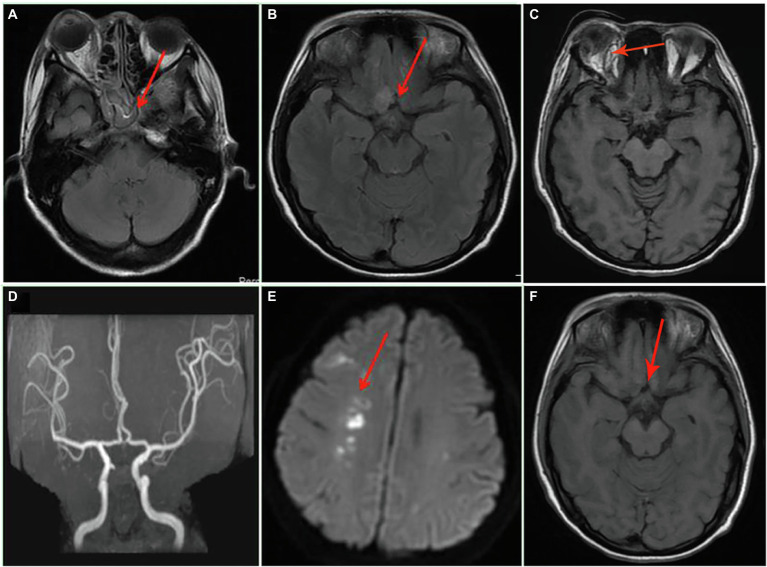
Axial MRI and magnetic resonance angiography of the brain on admission and after admission. **(A)** Inflammation of the sphenoid sinus and sinuses; **(B)** the frontal lobe lesion; **(C)** orbital MRI; **(D)** the normal magnetic resonance angiography; **(E)** the new cerebral infarction at the right basal ganglia 17 days after admission; and **(F)** the presentation of frontal lobe lesion after 67 days of treatment.

**Figure 2:**
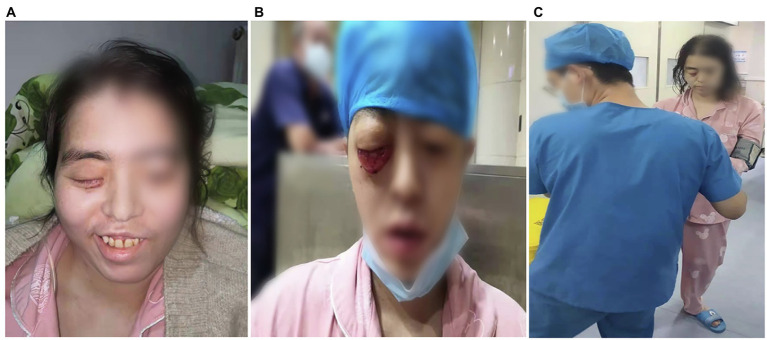
Facial features and limb muscle strength of the patient. **(A)** On admission, the patient presented with facial paralysis and askew mouth. After treatment, the conjunctival swelling had reduced. **(B)** On admission, the patient presented with right eye proptosis and fixation, conjunctival hyperemia and edema, and drooping eyelids **(C)** after 67 days of treatment, the patient’s limb muscle strength showed grade V.

A 34-year-old woman with right-sided headache for 8 days, blindness in the right eye with swelling for 5 days, and blindness in the left eye for 1 day was admitted to our hospital on July 13, 2021. Her medical history included left nephrectomy and macrosomia for three times. MRI showed the patient had sphenoid sinus, sinuses, and frontal lobe lesions (Figures 1A,B). Meanwhile, we observed thickening of the right eye ring, overstriking of the extraocular muscles and optic nerve, and proptosis of the right eye (Figure 1C). No abnormalities were observed *via* magnetic resonance angiography (Figure 1D). In terms of facial features, the patient presented with proptosis and fixation of the right eye, conjunctival hyperemia and edema, drooping eyelids, and askew mouth (Figures 2A,B). Additionally, blood and cerebrospinal fluid metagenomic sequencing (mNGS) revealed *Rhizopus oryzae* infection. In pertinent laboratory examinations, her glycated hemoglobin (HbA1c) was 17.8%, and fungus β-D-glucan was 240.64 pg/ml. Finally, she was diagnosed with ROCM and diabetes.

### Treatment

With input from the infectious disease service, the patient was initiated on intravenous ABCD (CSPC Ouyi Pharmaceutical Group Co., Ltd.) 6 mg/kg every 24 h, oral posaconazole (300 mg every 24 h), and topical amphotericin B (5 mg amphotericin B was dissolved in 30 ml normal saline and administered in three equally divided doses). Meanwhile, surgical debridement of sinus and bilateral and vitreous abscess (a symptom of fungal endophthalmitis) were performed several times on the day of admission and thereafter. In terms of blood sugar control, insulin has been actively used for treatment. After 67 days of treatment (with a cumulative dose of 21,550 mg for ABCD), the patient was discharged and transitioned to treatment with oral posaconazole (Kashyap et al., 2019).

### Surgical Procedure

Debridement of the sinuses and bilateral sphenoid sinuses referred to previous study (Hamilton et al., 2003). First, open the sinuses and pterygopalatine fossa. Next, the bilateral sphenoid sinuses were fused and the sinus secretions and mucous membranes were subsequently cleared. No bone destruction was found on the exposed bone surface. During the postoperative hospitalization, the above-mentioned parts were cleaned under the endoscope several times and washed with amphotericin B.

Evisceration was performed according to previous study (Murthy et al., 2022). Briefly, the conjunctiva was incised annularly along the limbus and found that there was no obvious infection foci in the retrobulbar tissue by pulling the extraocular muscle for examine. Then, the sclera was incised, and the sclera including the cornea was excised circularly to remove bulk of the purulent material in the ocular contents.

### Clinical Course

**Figure 3:**
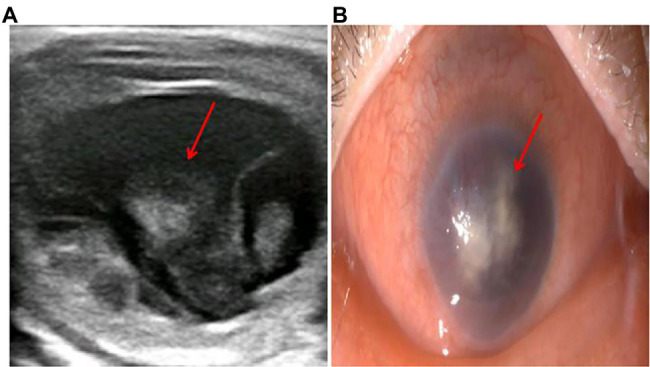
The diagnosis of vitreous abscess 50 days after admission. **(A)** Repeat ocular ultrasonography; **(B)** anterior segment photograph; the patient’s right eye showed severe vitreous opacity, incomplete posterior vitreous detachment, exudative retinal detachment (with numerous diffuse and weak echoes from the bulbar wall), bulbar wall thickening, and bulbar sac effusion.

**Figure 4:**
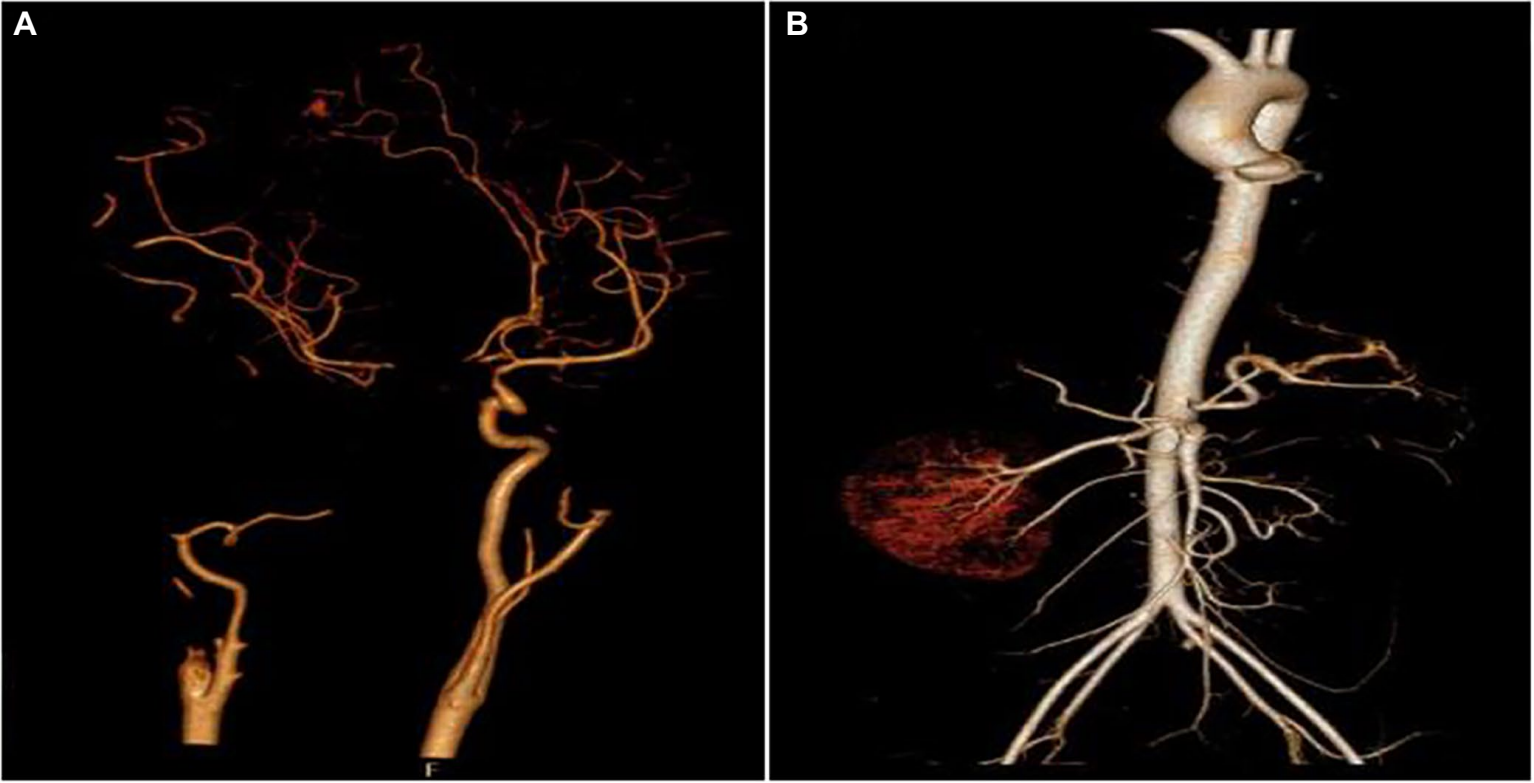
Aortic computed tomography angiography (CTA) of the cranial and abdominal artery 70 days after admission. **(A)** Right internal carotid artery and M1 middle cerebral artery were absent and **(B)** the left kidney and the left renal artery were absent.

After 17 days of hospitalization, the patient developed limb weakness, and repeat head MRI suggested multiple cerebral infarctions and was subsequently treated with aspirin (Figure 1E). After 50 days of treatment, repeat ocular ultrasonography (Figure 3A) and anterior segment photograph (Figure 3B) showed vitreous abscess, which was also the first definite photograph of vitreous abscess caused by mucormycosis. The patient’s right eye showed severe vitreous opacity, incomplete posterior vitreous detachment, exudative retinal detachment (with numerous diffuse and weak echoes from the bulbar wall), bulbar wall thickening, and bulbar sac effusion. Furthermore, evidence of vitreous abscess on polymerase chain reaction indicated *R. oryzae* infection. Considering the formation of vitreous abscess, the patient underwent evisceration to remove purulent secretions from the eye. In addition, during the procedure, no periorbital tissue necrosis was found. After 67 days of treatment, the patient’s condition was significantly improved, with reduced frontal lobe lesion and conjunctival swelling (Figures 1F, 2A), increased limb muscle strength (grade V; Figure 2C), and negative results for *R. oryzae*, although computed tomography angiography (CTA) showed the absence of right internal carotid artery and M1 middle cerebral artery (Figure 4A). Notably, no nephrotoxic events occurred during the treatment, even though the left kidney was resected (Figure 4B). The inpatient medicine team continued aggressive antifungal treatment as well as blood sugar control, lowering the HbA1c to 6.0%. After discharge, she was transitioned to oral posaconazole and was free of complaints in the 30-day follow-up without any additional treatment for ROCM. Unfortunately, the patient’s vision did not improve significantly.

## Discussion

The early symptoms of ROCM are atypical. When *Mucor* invades the sinuses, the patient will experience sinusitis-like symptoms; when the infection gradually spreads to the eyes, the patient will experience rapid exophthalmos, swelling of the eyelid, and loss of vision, suggesting mucormycosis (Galetta et al., 1990). In this case, the patient showed sphenoid sinus, sinuses, and frontal lobe lesions, accompanied by proptosis and fixation of the right eye, conjunctival hyperemia and edema, drooping eyelids, and askew mouth, indicating that the patient may had mucormycosis. A previous study showed that patients with mucormycosis are usually accompanied by diabetes, immunodeficiency, hormone application, and hematological malignancies (Honavar, 2021). Consistently, this patient presented with a high HbA1c of 17.8%. In addition, we noted that the fungal β-D-glucan of the patient was 240.64 pg/ml. *Mucor* is a conjugated bacterium whose pathogen does not produce the fungus β-D-glucan. Therefore, the β-D-glucan should theoretically be negative. However, in actual clinical work, existing data has shown that β-D-glucan was elevated in some patients with mucormycosis (Son et al., 2017). For this phenomenon, there was no relatively systematic report, and the reason was also unclear. However, we speculated that the possible causes include co-infection or false positives caused by treatment means. Early diagnosis and treatment are crucial (Karadeniz Uğurlu et al., 2015); however, only 33% of patients with mucormycosis could be identified by tissue culture. Given the false positive of G test and the incompleteness of tissue culture judgment, simultaneous gene sequencing would be a better complement because it allows faster and more efficient diagnosis (Bengel et al., 2007). Here, our mNGS result of blood and cerebrospinal fluid revealed that the patient was infected with *R. oryzae*, further confirming the diagnosis. According to the site of infection, ROCM can be divided into three types: paranasal, rhino-orbital, and ROC. Its overall mortality rate is higher than 50%, and the mortality rate after intracranial invasion is higher than 85% (Galletti et al., 2020). This case belongs to ROC and has the highest mortality rate, which should be paid more attention. *Mucor* is highly invasive to blood vessels and can cause tissue necrosis and blackening, which is a characteristic feature of ROCM (Yohai et al., 1994). Previously, Rangwala et al. (2019) reported a case of an intracranial aneurysm caused by pulmonary *Mucor*. In this case, the fungus was isolated from the blood and cerebrospinal fluid, while no obvious abnormality was observed in CTA of the thoracic region and abdominal vessels. However, it is important to note that even patients who improve clinically with antifungal therapy may continue to develop vascular complications (Little and Cheng, 2019). In the course of treatment, we found that the CTA of patient revealed the absence of right internal carotid artery and M1 middle cerebral artery, indicating the dynamics of vascular complications.

Amphotericin B, as a fungicide, is the preferred drug for the treatment of mucormycosis (Schwarz and Cornely, 2019). However, the occurrence of nephrotoxicity limits its usage. Conversely, increasing the dose of ABCD does not increase the adverse reaction. ABCD forms disc-shaped aggregates that are rapidly taken up by the organs with reticuloendothelial system, such as liver, spleen, and lung to form a temporary reservoir. After phagocytosis, the ABCD complex dissociates into amphotericin B and then released, which makes ABCD less nephrotoxic than amphotericin B (Herbrecht et al., 2001). The patient in this case was treated with ABCD for 67 days with a cumulative dose of 21,550 mg. Even at this high dose and the patient’s single kidney, no nephrotoxicity occurred during treatment, confirming the advantage of ABCD in terms of low nephrotoxicity. Posaconazole is a triazole antifungal drug that can inhibit ergosterol biosynthesis on the fungal cell membrane (Schwarz and Cornely, 2019). It has been reported that when combined with posaconazole, ABCD can achieve a better therapeutic effect (Ibrahim et al., 2008). Encouragingly, in our case, ABCD combined with posaconazole for the antifungal treatment of ROCM achieved a good therapeutic effect, and the patient was free of complaints in the 30-day follow-up after she was transitioned to oral posaconazole, without any additional treatment for mucormycosis.

Surgical debridement and drainage can reduce the level of fungus and help to control the disease progression. Herbrecht et al. (2001) showed that although the overall response rates were similar in patients treated with ABCD alone and patients treated with both ABCD and surgery debridement, the higher complete response rate in the latter group suggests that aggressive and sometimes repeated surgical removal of the infected necrotic tissue may be beneficial. The pterygopalatine fossa communicates with the surrounding tissues through eight channels and is an important hub for disease progression. Meas et al. (2007) suggested that exposure of the pterygopalatine fossa could improve patient survival. Hence, in this case, after exposure of the pterygopalatine fossa and repeated debridement and drainage operations, the patient preserved the periorbital tissue and achieved a good outcome.

There is increasing evidence of the importance of topical therapy in the treatment of mucormycosis. For example, Vironneau et al. (2014) showed a statistically significant difference in mortality between patients who received topical therapy or without. Navarro-Perea et al. (2019) presented a case of improvement with surgery combined with local therapy and emphasized the importance of topical management. Joos and Patel (2017) also proposed conservative orbital debridement with local antifungal irrigation is a viable option as well. Therefore, based on the better clinical results of the above local treatments, different local treatments to different lesions of the patients were performed. However, the treatment was eventually abandoned because the patient could not tolerate intravitreal injections. Additionally, it is worth noting that the periorbital tissue of this patient was intact, even if vitreous abscess was formed, indicating that local treatment was still effective and important. Roden et al. (2005) reviewed 929 patients with mucormycosis and found that diabetes was an independent risk factor for ROCM, emphasizing the importance of intensive insulin therapy. During the treatment, this patient has been actively controlling her blood sugar through insulin, thereby lowering the HBA1c to 6.0%.

## Conclusion

In summary, the duration of antifungal therapy to cure mucormycosis is unknown. Control of the underlying disease, immune status of the body, and adequate control of the source of infection are the key factors to be considered when deciding to stop treatment. Patients with one or more risk factors may require extended treatment or even lifelong treatment. Here, we successfully treated a case of ROCM with fungal endophthalmitis in which ABCD exhibited acceptable nephrotoxicity. This regimen can be considered as a promising option for this deadly disease.

## Data Availability Statement

The raw data supporting the conclusions of this article will be made available by the authors, without undue reservation.

## Ethics Statement

This study was approved by the Ethics Committee of The Second Hospital of Hebei Medical University. Written informed consent was obtained from the individual for the publication of any potentially identifiable images or data included in this article.

## Author Contributions

YZ and WT conceived of the study. JY, XL, HL, NY, PZ, CL, PC, and GL contributed to data collection, analysis, and interpretation of the results. YL wrote the manuscript. All authors contributed to the article and approved the submitted version.

## Conflict of Interest

The authors declare that the research was conducted in the absence of any commercial or financial relationships that could be construed as a potential conflict of interest.

## Publisher’s Note

All claims expressed in this article are solely those of the authors and do not necessarily represent those of their affiliated organizations, or those of the publisher, the editors and the reviewers. Any product that may be evaluated in this article, or claim that may be made by its manufacturer, is not guaranteed or endorsed by the publisher.
